# New Horizons in Metabolic Health: Unveiling the Future of Drug Discovery and Development

**DOI:** 10.2174/0118715303453651260321115118

**Published:** 2026-04-13

**Authors:** Jinshan Zhao, Quanyu Qiu, Jidong Zhang, Jun Tan

**Affiliations:** 1 Department of Histology and Embryology, Zunyi Medical University, Zunyi 563000, China;; 2 The First Clinical College, Zunyi Medical University, Zunyi 563000, China;; 3 Department of Immunology, Zunyi Medical University, Zunyi 563000, China

**Keywords:** Metabolic diseases, drug innovation, multi-target therapy, therapeutic drugs, glycemic control, SGLT2 inhibitors

## Abstract

**Introduction:**

Metabolic diseases (diabetes, obesity, NAFLD) pose a severe threat to global health, demanding innovative drug solutions.

**Methods:**

This review systematically summarizes studies (2018–2025) on metabolic disease drug targets, innovative drug classes, and technological advancements, retrieved from PubMed, Embase, and Web of Science.

**Results:**

Key targets include PPARs (improving insulin resistance); AMPK (regulating energy metabolism); and gut microbiota-derived SCFAs; innovative drugs such as GLP-1 RAs (liraglutide) and SGLT2 inhibitors (dapagliflozin) show efficacy in glycemic control and liver protection; AI and CRISPR accelerate drug development.

**Discussion:**

Challenges include interindividual microbiota variability and long-term safety of new drugs.

**Conclusion:**

Target discovery, drug innovation, and technological progress offer new hope for metabolic disease treatment, with a future focus on multi-target therapy and precision medicine.

## 1 INTRODUCTION

Metabolic diseases are a group of disorders caused by abnormal metabolic processes in the body, encompassing various types such as diabetes, obesity, hyperlipidemia, and non-alcoholic fatty liver disease (NAFLD). With global economic development and changes in lifestyle, the incidence of metabolic diseases has been increasing year by year, posing a severe threat to human health and quality of life, and imposing a heavy economic burden on society [1].

Take diabetes as an example: the global number of diabetes patients continues to grow, with an expected increase to 783 million by 2045 [2]. The situation of obesity is also severe. As of 2023, the overall obesity prevalence rate among children and adolescents was 8.5% [3]. Compared with the period from 2000 to 2011, the obesity prevalence rate increased by 1.5 times from 2012 to 2023 [3]. The incidence of non-alcoholic fatty liver disease (NAFLD) is significantly increasing. The predicted prevalence rate in 2040 is 55.7%, which has tripled since 1990 and increased by 43.2% compared to the prevalence rate of 38.9% in 2020 [4].

Traditional therapeutic drugs for these diseases often suffer from limited efficacy and significant side effects. Therefore, there is an urgent need for innovative drugs to improve treatment outcomes and patient prognosis. In recent years, with continuous research advancements, significant progress has been made in drug development for metabolic diseases in terms of target discovery, drug type innovation, and research technology innovation, bringing new hope for conquering metabolic diseases.

## 2 METHODOLOGY

To ensure the comprehensiveness, objectivity, and reliability of this review on metabolic disease drug innovation, a systematic approach was adopted for literature search, study selection, and data synthesis, strictly following the PRISMA standards to minimize bias and ensure transparency. Literature search covered major English databases such as PubMed, Embase, and Web of Science Core Collection. Search terms were combined around three core dimensions: “metabolic diseases”, “drug innovation”, and “mechanisms/technologies” (*e.g*., “diabetes OR obesity OR NAFLD” AND “drug targets OR novel drugs OR gene editing”). Additionally, references of included studies were manually screened, and abstracts from international conferences were supplemented to obtain the latest unpublished data.

Study selection was independently conducted by two researchers (J.Z., Q.Q.) in three steps: first, excluding irrelevant studies (*e.g*., non-metabolic diseases, pure physiological research) *via* titles/abstracts; second, screening full texts based on inclusion criteria (studies on target discovery, new drug development, or technology application with complete data) and exclusion criteria (methodological flaws, duplicate publications); third, assessing quality through discussion or arbitration by a professional third researcher (J.T.). A standardized Excel form was used for data extraction, collecting basic study information, core content, key results, and limitations, with cross-validation after extraction. Due to high heterogeneity in study types and outcome indicators, a narrative synthesis approach was adopted. Data were integrated around three themes—“therapeutic targets”, “novel drugs”, and “technological innovation”—to summarize consistent conclusions and controversies, forming a logically coherent evidence chain.

## 3 RESULTS

### 3.1 Discovery in Metabolic Disease Drug Target

#### 3.1.1 Targets Related to Insulin Resistance

Insulin resistance is one of the core pathological mechanisms of many metabolic diseases, such as type 2 diabetes and obesity. In-depth research on the occurrence and development of insulin resistance helps identify new therapeutic targets. The peroxisome proliferator-activated receptor (PPAR) family is closely associated with insulin resistance. The PPAR family includes three subtypes: PPARα, PPARβ/δ, and PPARγ, which play important roles in energy metabolism and immune responses [5]. The roles of PPARα, PPARβ/δ, and PPARγ in energy metabolism are shown in Fig. (**1**). Among them, PPARγ is crucial for adipocyte differentiation and lipid metabolism and is closely linked to insulin sensitivity. Activation of PPARγ can improve insulin sensitivity by inhibiting inflammatory mediators such as tumor necrosis factor-α (TNF-α) and interleukin-6 (IL-6) [6]. Additionally, the role of PPARγ in obesity and insulin resistance has been extensively studied. Obesity is often accompanied by chronic low-grade inflammation, and PPARγ can alleviate this inflammatory state by regulating adipocyte differentiation and lipid metabolism, thereby improving insulin resistance [7-9]. Synthetic ligands of PPARγ, such as thiazolidinediones, have been used in the treatment of type 2 diabetes [10]. The roles of PPARα and PPARβ/δ in insulin resistance should not be overlooked. PPARα is mainly expressed in the liver and participates in fatty acid oxidation and energy metabolism [11, 12]; its activation can improve lipid metabolism disorders and exert positive effects on insulin resistance [13, 14]. PPARβ/δ plays a role in regulating fatty acid oxidation and energy expenditure, and its activation may help alleviate obesity-related metabolic disorders [15-17]. For more specific comparisons of PPARα, PPARβ/δ, and PPARγ, please refer to Table **1**. Currently, the development of drugs that can act on multiple PPAR subtypes simultaneously to achieve more comprehensive metabolic regulation has become a new research direction, such as the development of PPAR pan-agonists, which has attracted significant attention. Research on insulin resistance and the PPAR family should not be limited to drug development, but also requires attention to the integration of lifestyle interventions with molecular mechanism studies. In-depth exploration of the effects of non-pharmacological measures such as exercise and dietary structure adjustment on PPAR expression and activity may provide more universally applicable solutions for the prevention and treatment of metabolic diseases.

Protein tyrosine phosphatase 1B (PTP1B) is another important target related to insulin resistance. PTP1B inhibits the insulin signaling cascade by dephosphorylating the insulin receptor and its substrates, leading to the development of insulin resistance [18]. Therefore, inhibiting PTP1B activity is considered a potential strategy for treating type 2 diabetes [19]. Studies have shown that PTP1B inhibitors can reduce blood glucose levels by enhancing insulin receptor sensitivity and help improve insulin resistance-related diseases [20]. Furthermore, PTP1B deficiency is associated with the restoration of leptin and insulin sensitivity in obese mice. Research has found that mice lacking PTP1B exhibit enhanced leptin and insulin signaling in the hypothalamus, thereby suppressing appetite, increasing brown fat formation in adipose tissue, reducing fat mass, and improving glucose metabolism [21]. Another study designed and synthesized 1,3,4-thiadiazolyl thiazolidine-2,4-dione derivatives as PTP1B inhibitors. The results showed that these compounds not only exhibited potential PTP1B inhibitory activity but also improved insulin resistance, reduced blood glucose levels, and ameliorated glucose tolerance and dyslipidemia [22].

### 3.2 Energy Metabolism Regulatory Targets

#### 3.2.1 Adenosine Monophosphate-activated Protein Kinase (AMPK) Pathway

Adenosine monophosphate-activated protein kinase (AMPK) is a key regulator of cellular energy metabolism. When the intracellular AMP/ATP ratio increases, AMPK is activated, regulating a series of downstream target proteins to promote glucose uptake and fatty acid oxidation while inhibiting fat synthesis and gluconeogenesis, thus maintaining cellular energy balance. In metabolic disease states, the activity of AMPK (5-adenosine monophosphate-activated protein kinase) is often suppressed, and activating AMPK can significantly improve insulin sensitivity. This mechanism has been validated in multiple studies. For example, MK-8722, a systemic AMPK activator, has been shown to promote glucose uptake and glycogen synthesis in skeletal muscle by activating AMPK in rodents and rhesus monkeys, thereby improving blood glucose levels [23]. Additionally, the water extract of *Scrophularia ningpoensis* has been demonstrated to enhance insulin sensitivity through AMPK-mediated inhibition of the NLRP3 inflammasome [24]. In obesity and chronic diseases, the role of AMPK has also been widely studied. AMPK is inhibited in many pathological states, such as inflammation, diabetes, aging, and cancer, while its activation has positive effects in improving insulin resistance, diabetes, obesity, and other diseases [25].

These studies indicate that activating AMPK through nutritional regulation and drug intervention can serve as a potential strategy for treating metabolic diseases [26]. Furthermore, research has found that the root bark extract of *Broussonetia papyrifera* (PRE) improves glucose tolerance and reduces inflammation in adipose tissue of high-fat diet-induced obese mice by activating AMPK [27]. Similar to PRE, other compounds such as elaiophylin and pumpkin polysaccharide (PPS3) have been shown to activate AMPK, thereby improving metabolic outcomes in obese mice. For instance, elaiophylin reduces body weight and glucose levels by increasing AMPK activity, while PPS3 enhances glucose tolerance and lipid metabolism [28, 29]. These findings further support the view of AMPK as a key target for treating metabolic diseases. Activating AMPK not only improves insulin sensitivity but also regulates lipid metabolism and reduces inflammation, providing new insights for the treatment of metabolic diseases. Studies on AMPK have shown that activating AMPK can significantly improve insulin sensitivity, which holds great potential in the treatment of metabolic diseases [27]. In practical clinical settings, the precise regulation of AMPK activity *via* nutritional modulation and drug intervention represents a critical focus for future research in the field [30, 31]. Through personalized treatment, unnecessary side effects can be avoided, and treatment effects can be optimized.

#### 3.2.2 Uncoupling Protein (UCP) Pathway

Another target closely related to energy metabolism is the uncoupling protein (UCP) family, which consists of five members (UCP1-UCP5) with distinct tissue distributions and biological functions [32]. Among them, UCP1 is mainly found in brown adipose tissue and can uncouple the oxidative phosphorylation process, releasing stored chemical energy as heat to increase energy expenditure, which is crucial for maintaining energy balance and body temperature regulation [33]. By forming proton channels in the inner mitochondrial membrane, UCP1 dissipates the proton gradient as heat (non-shivering thermogenesis), thereby increasing energy expenditure and contributing to body temperature regulation and energy balance maintenance [34]. In patients with obesity and type 2 diabetes, the function of brown adipose tissue (BAT) is often impaired, which is closely related to reduced expression and activity of UCP1 (uncoupling protein 1) [35]. Studies have shown that obesity and a high-fat diet lead to decreased UCP1 expression in brown adipose tissue, affecting its thermogenic capacity [36]. Additionally, in the brown adipose tissue of obese patients, increased expression of TRB3 protein is associated with decreased UCP1 expression, further contributing to impaired brown adipose tissue function [36].

Beyond UCP1, UCP2 and UCP3 also play important roles in energy metabolism and metabolic disease progression. UCP2 is widely expressed in tissues such as the liver, pancreas, and adipose tissue, and is involved in regulating reactive oxygen species (ROS) production, lipid metabolism, and insulin secretion [37-39]. UCP2 knockout mice exhibit increased insulin sensitivity and resistance to HFD-induced obesity, while UCP2 overexpression is associated with impaired insulin secretion in pancreatic β-cells [40]. UCP3, mainly expressed in skeletal muscle and BAT, participates in fatty acid transport and oxidation, and its downregulation in skeletal muscle of obese individuals contributes to lipid accumulation and insulin resistance [41, 42].

In the context of obesity and type 2 diabetes, the activity and mass of brown adipose tissue are significantly reduced, which may be due to the combined effects of multiple pathological processes, including catecholamine resistance, inflammation, oxidative stress, and endoplasmic reticulum stress [43]. These factors collectively affect the normal differentiation and function of brown adipose tissue, leading to decreased thermogenic capacity and adversely impacting overall energy expenditure and body weight regulation. Considering the core role of UCP1 in energy metabolism, activating UCP1 expression or enhancing its activity may be incorporated into therapeutic strategies in the future. Future research should focus on elucidating the molecular mechanisms underlying UCP regulation and identifying novel targets for UCP activation. Additionally, combining UCP-targeted therapies with AMPK activators or other metabolic regulators may provide synergistic effects in improving metabolic diseases, offering new avenues for personalized treatment.

### 3.3 Gut Microbiota-Related Targets

#### 3.3.1 Short-Chain Fatty Acids (SCFAs)

In recent years, growing evidence has indicated that gut microbiota dysregulation is closely linked to metabolic disorders such as insulin resistance, obesity, and NAFLD. Short-chain fatty acids (SCFAs), key metabolites derived from the gut microbial fermentation of dietary fiber—primarily including acetate, propionate, and butyrate—not only serve as energy substrates for the host but also modulate energy metabolism, insulin secretion, and inflammatory responses through activation of G protein-coupled receptors (*e.g*., GPR41, GPR43) on intestinal endocrine cells. Murine studies have shown that propionate can lower blood glucose levels by suppressing hepatic gluconeogenesis, thereby ameliorating metabolic syndrome [44]. The mechanism of action of propionic acid may be related to its metabolites in the gut, which can regulate the composition and function of the gut microbiota, thereby indirectly influencing hepatic gluconeogenesis [45]. On the other hand, butyric acid is renowned for its anti-inflammatory properties and can improve insulin resistance by reducing the activity of inflammatory factors such as nuclear factor κB [46]. The anti-inflammatory effects of butyric acid extend beyond the liver to skeletal muscle and adipose tissue, which also play important roles in metabolic regulation [47]. Additionally, butyric acid can further improve insulin sensitivity by regulating fatty acid oxidation and lipid metabolism [46]. From an application perspective, dietary interventions such as increasing dietary fiber intake or supplementing with SCFAs preparations to regulate gut microbiota metabolism provide new strategies for the prevention and treatment of metabolic diseases. However, many challenges remain: the significant structural and functional differences in gut microbiota among individuals lead to interindividual variability in SCFAs production and response.

#### 3.3.2 Bile Acids

The role of bile acids in fat digestion and absorption has been widely studied. However, recent research has shown that bile acids can also act as signaling molecules to regulate various metabolic processes by binding to receptors such as the farnesoid X receptor (FXR) and G protein-coupled bile acid receptor 1 (TGR5) [48, 49]. FXR, the primary nuclear receptor for bile acids, is predominantly expressed in the liver and intestine. It maintains bile acid homeostasis by regulating bile acid synthesis and enterohepatic circulation [50]. Additionally, FXR plays a critical role in glucose and lipid metabolism. Activation of its signaling pathway can reduce lipogenesis and inhibit gluconeogenesis, thereby alleviating metabolic diseases [48].

On the other hand, TGR5, a membrane-bound bile acid receptor, is mainly expressed in the intestine. Activation of TGR5 can reduce inflammatory responses by inhibiting NFκB and improve insulin sensitivity by inducing enterocytes to secrete glucagon-like peptide-1 (GLP-1) [51]. Furthermore, TGR5 is involved in regulating energy and lipid metabolism. Studies have found that TGR5 activation can improve obesity and related metabolic disorders by promoting fatty acid oxidation and energy expenditure [52]. At present, the mechanisms of synergistic action between FXR and TGR5 under different physiological and pathological conditions remain unclear. In the future, it will be necessary to conduct in-depth research on the interactive regulatory network between the two, which is crucial for a comprehensive understanding of the role of bile acid signaling pathways in metabolic regulation and the development of more precise and effective therapeutic strategies for metabolic diseases.

#### 3.3.3 Indolepropionic Acid (IPA) and Aryl Hydrocarbon Receptor (AHR)

Indolepropionic acid (IPA), a metabolite of tryptophan produced by gut microbiota, plays a critical role in activating the aryl hydrocarbon receptor (AHR) and improving insulin sensitivity. Research in mice has shown that IPA acts as an endogenous leptin sensitizer and can counteract diet-induced obesity by targeting STAT3. This process involves IPA being secreted from the gut into the circulatory system, promoting phosphorylation and nuclear translocation of STAT3 in the hypothalamic appetite regulatory center, thereby enhancing leptin responsiveness and regulating appetite and energy metabolism balance [53].

The mechanisms by which AHR (aryl hydrocarbon receptor) activation influences insulin sensitivity involve multiple signaling pathways and molecular processes. On one hand, AHR activation can reduce insulin sensitivity by promoting inflammation and lipid metabolism disorders. For example, gut microbiota-derived metabolites such as kynurenine (Kyn) disrupt lipid metabolic homeostasis in adipocytes and exacerbate lipid deposition within adipocytes through activating the AHR/STAT3/IL-6 signaling pathway, thereby decreasing systemic insulin sensitivity [54]. Additionally, AHR activation further exacerbates insulin resistance by upregulating the expression of inflammatory cytokines such as TNF-α, IL-6, and IL-1β [55].

On the other hand, AHR activation can also improve insulin sensitivity by regulating specific metabolic pathways. For instance, gut microbiota-derived metabolites like 5-HIAA directly activate AHR to promote hepatic insulin signaling by stimulating TSC2 transcription, thereby inhibiting mTORC1 signaling and improving glucose intolerance and insulin sensitivity [56, 57]. Furthermore, AHR activation is involved in regulating insulin sensitivity and adipose tissue browning by modulating the expression of fibroblast growth factor 21 (FGF21) [57]. From a clinical application perspective, developing therapeutic strategies to improve insulin sensitivity based on IPA and AHR holds broad prospects but also faces numerous challenges. On one hand, there is the question of how to precisely modulate the gut microbiota to increase IPA production while avoiding the generation of other harmful metabolites. On the other hand, the development of AHR agonists or antagonists must take into account their bidirectional effects on insulin sensitivity to prevent new metabolic disorders caused by excessive activation or inhibition of AHR.

### 3.4 The Apelin/APJ Pathway

The Apelin-13/APJ system is a key signaling axis in the fields of cardiovascular and metabolic physiology, composed of the peptide ligand Apelin-13 and its G protein-coupled receptor (GPCR), APJ. This system plays a critical role in regulating appetite, vascular tone, and energy metabolism. Studies have shown that Apelin-13, as an endogenous ligand for the APJ receptor, significantly enhances insulin sensitivity through binding to this receptor, thereby improving obesity-related metabolic disorders and cardiac dysfunction [58]. In the context of obesity and metabolic syndrome, the role of Apelin-13 is particularly notable: it not only promotes glucose utilization but also improves insulin resistance by regulating the function of adipose tissue [59].

In obese individuals, Apelin-13 improves insulin-stimulated vasodilation by increasing nitric oxide production and attenuates vasoconstriction mediated by angiotensin II and endothelin-1 [60]. In terms of energy metabolism, Apelin-13 influences whole-body energy balance by regulating metabolic activities in adipose tissue, promoting fat breakdown and utilization [61]. Research indicates that Apelin-13 can activate the AMPK pathway to inhibit fatty acid synthesis and promote fatty acid oxidation, thus exerting therapeutic effects in metabolic diseases such as obesity and diabetes [62]. The Apelin-13/APJ system acts as a central regulator of cardiovascular homeostasis and metabolic balance through multi-tissue and multi-organ signaling. Its dysregulation is associated with multiple major diseases, while drug development targeting this system has opened new avenues for the treatment of cardiovascular and metabolic disorders. Future research could further explore the dynamic change patterns of the Apelin-13/APJ system under different pathological conditions, deeply excavate its interaction mechanisms with other signaling pathways, and accelerate the development of efficient and safe targeted drugs, which is expected to open up new avenues for the prevention and treatment of cardiovascular and metabolic diseases.

### 3.5 Innovative Drug Types for Metabolic Diseases

#### 3.5.1 Glucagon-Like Peptide-1 (GLP-1) Receptor Agonists

GLP-1 receptor agonists (GLP-1 RAs), which mimic the effects of native GLP-1 by binding to GLP-1 receptors, have been widely used in the treatment of type 2 diabetes and obesity. They also show potential in improving metabolic dysfunction-associated steatotic liver disease (MASLD). Studies have found that GLP-1 RAs such as liraglutide and semaglutide have certain efficacy in managing MASLD [63]. These drugs have demonstrated positive effects in reducing liver fat content, improving liver function indices (such as transaminase levels), and alleviating liver inflammation and fibrosis [64, 65].

In clinical applications, multiple GLP-1 RAs have been marketed, including liraglutide, exenatide, and dulaglutide. Liraglutide, semaglutide, and tirzepatide have been shown in clinical trials to significantly reduce liver fat content and improve liver fibrosis scores [65, 66]. As treatments for type 2 diabetes, liraglutide and dulaglutide have both demonstrated significant effects in lowering blood glucose and body weight. Liraglutide, a GLP-1 RA, can effectively reduce glycated hemoglobin (HbA1c) levels while significantly reducing patient body weight. In one study, liraglutide was proven to have good effects in lowering HbA1c and body weight, particularly when used in combination with other hypoglycemic drugs [67]. Additionally, long-term use of liraglutide is associated with good blood glucose and weight control [68]. Dulaglutide, also a GLP-1 RA, has shown favorable effects in reducing HbA1c and body weight. In one study, dulaglutide significantly reduced HbA1c levels across different body mass index (BMI) categories, indicating consistent efficacy regardless of baseline weight [69].

Furthermore, GLP-1 RAs indirectly improve the metabolic status of MASLD by enhancing insulin sensitivity, reducing body weight, and lowering blood glucose and lipid levels [64-65]. However, while clinical studies in humans have shown promising effects of GLP-1 RAs in improving steatosis and inflammation, their direct impact on liver fibrosis still requires further verification [64, 66]. There is still broad space for research on GLP-1 RAs in the field of MASLD treatment. In addition to further verifying their effect on improving liver fibrosis, the synergistic effects of combination therapy with other new drugs, as well as the safety and efficacy in different populations (such as children, the elderly, and pregnant women), can also be explored.

#### 3.5.2 Sodium-Glucose Cotransporter 2 (SGLT2) Inhibitors

SGLT2 inhibitors are a new class of oral hypoglycemic drugs that lower blood glucose levels by inhibiting sodium-glucose cotransporter 2 (SGLT2) in the renal proximal tubule, increasing urinary glucose excretion. This mechanism not only helps patients achieve better blood glucose control but also leads to weight loss. One reason for weight loss is energy loss due to urinary glucose excretion [70].

Currently, SGLT2 inhibitors such as dapagliflozin, empagliflozin, and canagliflozin have been widely used in clinical practice. Dapagliflozin not only effectively lowers blood glucose levels but also has significant renal protective effects. For instance, research in mice has demonstrated that dapagliflozin can improve diabetic nephropathy by inhibiting ferroptosis and promoting β-hydroxybutyrate production [71]. Furthermore, when combined with telmisartan, dapagliflozin significantly reduces urinary albumin excretion rate and inflammatory marker levels, thereby improving renal function in diabetic nephropathy patients [72]. Dapagliflozin also exhibits positive effects on cardiovascular protection. Studies in humans have shown that it provides cardiac and renal protection in patients by improving energy metabolism, reducing inflammatory activity, and decreasing insulin resistance [73]. Additionally, dapagliflozin has demonstrated significant effects in reducing body weight and blood pressure. In one study, dapagliflozin treatment significantly reduced patient body weight and blood pressure, and this effect was independent of changes in dietary intake or behavior [74]. The advent of SGLT2 inhibitors marks a strategic upgrade in diabetes treatment from mere “blood glucose control” to “metabolic remodeling”. Their core value lies not only in achieving “passive glucose clearance” through urinary glucose excretion but also in activating the body's own energy homeostasis regulation mechanism, thereby enabling healthier body composition remodeling. This stands in stark contrast to the central appetite suppression mechanism of traditional weight-loss drugs.

#### 3.5.3 Fatty Acid-Binding Protein (FABP) Inhibitors

FABPs are a group of proteins that play important roles in intracellular fatty acid uptake, transport, and metabolism [75, 76]. Different FABP subtypes are distributed in various tissues: for example, liver-type FABP (FABP1) is mainly found in the liver and renal proximal tubules, while adipocyte-type FABP (FABP4) is predominantly expressed in adipose tissue, macrophages, and other cells [77-79]. In metabolic disease states, abnormal expression and function of FABPs are involved in pathological processes such as lipid metabolism disorders, insulin resistance, and inflammatory responses [75, 76, 80]. FABP4 (adipocyte-type) and FABP5 (epidermal-type) exhibit synergistic effects in lipid transport and metabolism [79]. Studies in mice have found that simultaneous inhibition of FABP4 and FABP5 can significantly improve lipid metabolism disorders induced by a high-fat diet by reducing the abnormal accumulation of lipids in adipocytes and macrophages [81]. For more comparisons of FABPs subtypes, please refer to Table **2**.

Currently, research and development of FABP inhibitors have made certain progress. The potential of FABP inhibitors in the treatment of metabolic diseases has attracted widespread attention. In recent years, researchers have developed FABP inhibitors through various strategies and achieved preliminary results in clinical trials. For example, cobimetinib was identified as a novel FABP inhibitor through virtual screening using machine learning and molecular docking methods; it significantly inhibits the activation of the JNK/c-Jun signaling pathway in mouse macrophages without causing obvious cytotoxicity [82].

Furthermore, the development of FABP/PPAR multi-modulators has provided new ideas for the treatment of metabolic diseases. Researchers have designed the first class of FABP/PPAR multi-modulators, which showed better therapeutic effects than the clinical candidate drug obeticholic acid in a metabolic dysfunction-associated steatohepatitis (MASH) mouse model [83]. The successful discovery of this multi-target drug provides preliminary evidence for the application of FABP inhibitors in metabolic diseases. The success of FABP/PPAR multi-modulators has indeed brought new hope for the treatment of metabolic diseases, but their mechanism of action still needs to be further clarified. Will such multi-target drugs disrupt the body's original homeostatic balance and trigger new metabolic disorders when regulating metabolic pathways? How to optimize drug design to maximize therapeutic effects while minimizing interference with normal physiological functions? Answering these questions will help FABP-related drug development move towards a safer and more effective direction.

#### 3.5.4 Drugs Targeting Hepatic Fat Synthesis

Abnormally increased hepatic fat synthesis is an important pathological feature of metabolic diseases such as NAFLD. Fatty acid synthase (FASN) is a key enzyme in hepatic fatty acid synthesis, catalyzing the synthesis of fatty acids from acetyl-CoA and malonyl-CoA; its overexpression is closely associated with the development of metabolic diseases such as non-alcoholic fatty liver disease (NAFLD). Studies have shown that inhibiting the activity of fatty acid synthase (FASN) can effectively reduce hepatic fat deposition and improve liver function indicators, with diverse mechanisms of action [84]. Studies in rats have demonstrated that, on one hand, alpha-lipoic acid can inhibit FASN expression by upregulating miR-3548 to reduce hepatic lipid accumulation; on the other hand, microRNA-103 can target FASN and SCD1 to inhibit hepatic de novo lipid synthesis, thereby alleviating the progression of non-alcoholic fatty liver disease (NAFLD) [85, 86]; and miR-30c-5p can reduce triglyceride accumulation in mice and cells by inhibiting FASN expression, thereby improving steatosis [87]. On the other hand, inhibition of O-GlcNAcylation can reduce hepatic lipid accumulation by promoting FASN ubiquitination and degradation, while FASN inhibitors can regulate cellular energy metabolism by activating the AMPK and inhibiting the mTORC1 signaling pathways [88]. Additionally, FASN expression is regulated by multiple signaling pathways such as ChREBP, SREBP-1c, and FXR/SHP/LXRα, which further modulate fatty acid synthesis and steatosis by influencing FASN transcription and translation [89, 90]. Clinical trials have shown that FASN inhibitors (such as TVB-2640) can reduce hepatic fat content and improve liver function indicators [91]. The critical role of FASN in hepatic fat deposition and its close association with steatosis establish it as an important therapeutic target for NAFLD, and FASN inhibitors hold potential application value in the treatment of NAFLD and improvement of liver function.

#### 3.5.5 Dipeptidyl Peptidase-4 Inhibitors (DPP-4i)

DPP-4 inhibitors (DPP-4i) play a crucial role in diabetes management. By suppressing the activity of dipeptidyl peptidase-4 (DPP-4), these agents increase the levels of glucagon-like peptide-1 (GLP-1) and glucose-dependent insulinotropic polypeptide (GIP). This mechanism promotes insulin secretion while inhibiting glucagon release, ultimately achieving glucose-dependent, precise glycemic control [92, 93]. As a result, DPP-4i demonstrate significant efficacy in reducing both fasting and postprandial blood glucose levels. Notably, their glucose-dependent action minimizes the risk of hypoglycemia [94].

Beyond glycemic control, DPP-4i exhibit multiple benefits extending beyond blood sugar regulation. For instance, studies in mice have shown that DPP-4i exert cardioprotective effects by alleviating diabetes-induced myocardial fibrosis and structural cardiac damage [95]. These drugs may also provide antioxidant and anti-inflammatory effects through mechanisms such as inhibiting cell apoptosis and oxidative stress, offering additional potential benefits in managing diabetic complications [96].

DPP-4i are not limited to monotherapy; they can be combined with other hypoglycemic agents to enhance therapeutic efficacy. For example, when used in conjunction with metformin, DPP-4i further improves islet function and insulin sensitivity, leading to more effective glycemic control [97]. This combination strategy is particularly important in East Asian populations, where islet dysfunction plays a pivotal role in the pathogenesis of type 2 diabetes [94]. Through the synergistic action of multiple mechanisms, DPP-4i provides comprehensive treatment options for patients with diabetes.

### 3.6 GLP-1/GIP Dual Receptor Agonists

GLP-1/GIP dual receptor agonists are a class of emerging drugs that exhibit more potent glucose-lowering, weight-loss, and metabolic-improving effects than single-receptor agonists by simultaneously activating both GLP-1 and GIP receptors. This dual mechanism of action not only enhances the stimulation of insulin secretion but also further promotes weight management by suppressing appetite and increasing energy expenditure [98, 99]. Studies have shown that GLP-1/GIP dual receptor agonists, such as tirzepatide have demonstrated significant weight loss effects in clinical trials and also possess remarkable advantages in improving metabolic parameters in patients with diabetes [100, 101]. Tirzepatide acts on the body through multiple mechanisms, including promoting insulin secretion, inhibiting glucagon release, delaying gastric emptying, *etc*., thereby effectively controlling blood glucose levels and facilitating weight management [102]. In the phase 2 clinical trial of another GLP-1/GIP dual receptor agonist, Mazdutide, after 24 weeks of once-weekly treatment, the subjects achieved an average weight loss of 6.7%, 10.4%, and 11.3%, respectively. In addition, in the 46-week phase 2 clinical trial of GLP-1/GCG dual receptor agonist Survodutide, the average weight loss of the subjects was 6.2%, 12.5%, 13.2%, and 14.9%, respectively. Moreover, in the 48-week phase 2 clinical trial of GLP-1/GIP/GCG triple receptor agonist Retatrutide, the average weight loss of the subjects was 17.1%, 22.8%, and 24.2%, respectively. These data indicate that dual receptor agonists have significant advantages in weight loss [103-105].

In the treatment of obesity, GLP-1/GIP dual receptor agonists have broad application prospects. These drugs can not only effectively reduce body weight but also improve lipid metabolism and insulin sensitivity, thereby reducing the risk of obesity-related metabolic diseases [106-107]. Although the short-term clinical trial results are encouraging, the long-term efficacy and safety of such new drugs still need further evaluation. For example, whether the long-term use of these drugs will have negative effects on important organs such as the liver and kidneys, or whether there are other potential side effects, are all issues that require further research.

### 3.7 Technological Innovations in Metabolic Disease Drug Development

#### 3.7.1 Application of Artificial Intelligence (AI) Technology

In target discovery, the application of artificial intelligence (AI) technology is rapidly transforming the landscape of drug development. AI can process and analyze large volumes of biological data, including genomics, proteomics, and metabolomics data, to identify potential drug targets. This capability has made AI play a significant role in drug target discovery.

One important application of AI in drug target discovery is the identification of new biomarkers and therapeutic targets through the integration of multi-omics data. By comprehensively analyzing genes, proteins, and metabolites, AI can reveal interaction networks at different molecular levels, providing in-depth insights into disease mechanisms. This approach has demonstrated great potential in the study of complex diseases such as diabetes [108].

AI can also predict drug-target interactions through machine learning models. These models can extract features from complex biological data, accurately model molecular interactions, and predict potential drug-target outcomes. This method not only improves the accuracy of drug target prediction but also accelerates the development of personalized medicine [109].

Another important application of AI in drug development is the identification of therapeutic targets through metabolomics-driven approaches. As a systems biology tool, metabolomics can capture phenotypic changes induced by exogenous compounds, providing a valuable method for target identification. Combined with AI technology, metabolomics can better understand disease mechanisms and accelerate the development of targeted drugs [110].

#### 3.7.2 Gene Editing Technology

Gene editing technologies, particularly the CRISPR-Cas9 system, have made significant progress in the treatment of metabolic diseases in recent years. The emergence of these technologies has brought revolutionary breakthroughs in the treatment of hereditary metabolic diseases, offering the possibility of fundamentally curing these diseases. For example, the CRISPR-Cas9 technology has shown great potential in the treatment of metabolic diseases such as hyperlipidemia and diabetes [111].

The CRISPR-Cas9 system enables scientists to directly repair or replace disease-causing gene mutations through precise gene editing capabilities, thereby correcting the root causes of metabolic diseases at the molecular level. The application of this technology extends beyond single-gene genetic diseases to the treatment of complex multigenic diseases [112]. Furthermore, the continuous development of CRISPR-Cas9 technology, such as its integration with multi-omics technologies, further enhances its application potential in the treatment of metabolic diseases [113].

#### 3.7.3 Application of Nanotechnology in Drug Delivery

Nanotechnology has unique advantages in drug delivery for metabolic diseases, capable of improving drug pharmacokinetic properties, enhancing drug targeting and bioavailability, and reducing drug toxicity and side effects. In this field, lipid nanosystems are considered an effective treatment strategy. Lipid nanosystems include liposomes, solid lipid nanoparticles, nanostructured lipid carriers, nanoemulsions, microemulsions, and phospholipid complexes. The composition and properties of these systems have been thoroughly studied to improve their efficiency in the treatment of fatty liver disease [114]. Additionally, surface modification of nanocarriers is regarded as a potential method to enhance targeted drug delivery. By modifying the surface of nanocarriers, controlled drug release, enhanced penetration efficiency, and targeted drug delivery can be achieved [115].

## CONCLUSION

Metabolic diseases, including type 2 diabetes, obesity, and non-alcoholic fatty liver disease (NAFLD), remain a pressing global health challenge, with rising incidence rates (*e.g*., 783 million projected diabetes cases by 2045 and 55.7% NAFLD prevalence by 2040) imposing substantial individual and societal burdens. Traditional therapies, limited by inadequate efficacy and significant side effects, have underscored the urgent need for innovation—and this review synthesizes the transformative progress achieved in metabolic disease drug development, spanning target discovery, therapeutic innovation, and technological advancement.

A foundational breakthrough lies in the identification of precise therapeutic targets, which has reshaped our understanding of disease pathogenesis and guided drug design. Key discoveries include insulin resistance-related molecules such as the PPAR family (α, β/δ, γ) and PTP1B—where PPARγ activation improves insulin sensitivity by mitigating inflammation, and PTP1B inhibition enhances insulin signaling—to energy metabolism regulators like the AMPK pathway (a master controller of cellular energy balance) and the UCP family (critical for brown adipose tissue thermogenesis). Additionally, gut microbiota-associated targets (short-chain fatty acids, bile acids, and the IPA/AHR axis) link intestinal homeostasis to systemic metabolic health, while the Apelin/APJ pathway integrates cardiovascular and metabolic regulation. Together, these targets provide molecular anchors for developing therapies that address the root causes of metabolic dysfunction, rather than just symptoms.

Complementing target discovery, the development of novel drug classes has revolutionized clinical practice. GLP-1 receptor agonists (*e.g*., liraglutide, semaglutide) and SGLT2 inhibitors (*e.g*., dapagliflozin) have become cornerstones of treatment, lowering blood glucose, reducing body weight, and improving liver, renal, and cardiovascular outcomes. FABP inhibitors, FASN-targeting drugs (for hepatic steatosis), and DPP-4 inhibitors further expand options by tackling lipid dysregulation and islet dysfunction. Notably, dual (*e.g*., GLP-1/GIP) and triple receptor agonists (*e.g*., retatrutide) represent a paradigm shift, delivering superior weight loss and metabolic benefits by engaging multiple signaling axes—catering to the heterogeneous nature of metabolic diseases.

Technological innovations have accelerated every stage of drug development. Artificial intelligence (AI) processes multi-omics data to predict therapeutic targets and drug-target interactions, streamlining candidate identification. CRISPR-Cas9 gene editing offers potential for curing hereditary metabolic defects by correcting pathogenic mutations, while nanotechnology enhances drug delivery—improving bioavailability, tissue targeting, and safety, which is critical for addressing organ-specific pathologies (*e.g*., hepatic steatosis). These tools not only reduce R&D timelines but also enable more precise, mechanism-driven therapies.

## CHALLENGES AND FUTURE PROSPECTS

Despite the challenges in the drug development process for metabolic diseases, the future outlook remains full of hope and potential. A deep understanding of the complex pathogenesis of these diseases serves as the cornerstone for driving innovation. With the rapid development of high-throughput, multi-dimensional research technologies, such as single-cell sequencing, spatial transcriptomics, advanced proteomics, and metabolomics, we will be able to map the molecular landscape in disease states with unprecedented precision and depth. This will uncover critical signaling pathways, cell types, and molecular network dysregulation, thereby identifying more novel and potentially targetable therapeutic targets.

Technological innovation will be a key driving force behind breakthroughs in metabolic disease drug development. The increasingly mature application of Artificial Intelligence (AI) in drug target prediction, molecular design, drug screening, and clinical trial optimization will significantly enhance R&D efficiency and success rates. Gene editing technologies (such as CRISPR-Cas9), although still needing to overcome challenges related to safety and delivery, show immense potential in correcting inherited metabolic defects and developing gene therapies. Furthermore, advancements in nanotechnology will improve drug delivery systems, enabling more precise targeting, sustained or controlled release, thereby enhancing efficacy and reducing side effects.

Future research will increasingly focus on the systemic nature of metabolic diseases and the interactions between organs. For example, a deeper understanding of adipose tissue dysfunction, hepatic steatosis, insulin resistance, gut microbiota dysbiosis, chronic inflammation, and the complex links between these factors and cardiovascular, renal, and neurological complications will provide new avenues for developing combination therapies or multi-target drugs capable of intervening in multiple pathogenic processes simultaneously. Moreover, focusing on key nodes in the disease progression process, such as the development from insulin resistance to β-cell failure, or the progression from fatty liver to NASH and cirrhosis, will aid in developing disease-modifying drugs capable of slowing down or reversing disease progression. Looking further ahead, the management of metabolic diseases may transition from treating established disease to preventing disease onset. Through early risk prediction (based on genetic, epigenetic, microbiome, metabolomic, and lifestyle data), combined with precise molecular diagnostic and monitoring technologies, early intervention for high-risk populations may become possible in the future.

In summary, metabolic disease drug development stands at a new starting point driven by breakthroughs in basic research, enabled by frontier technologies, and cross-disciplinary integration. In the future, we can expect to see the emergence of more innovative drugs with novel mechanisms, better efficacy, improved safety, and the capability for precise individualized treatment, thereby more effectively addressing the severe global challenge of metabolic diseases and significantly improving patients' long-term prognosis and quality of life.

## Figures and Tables

**Fig. (1):**
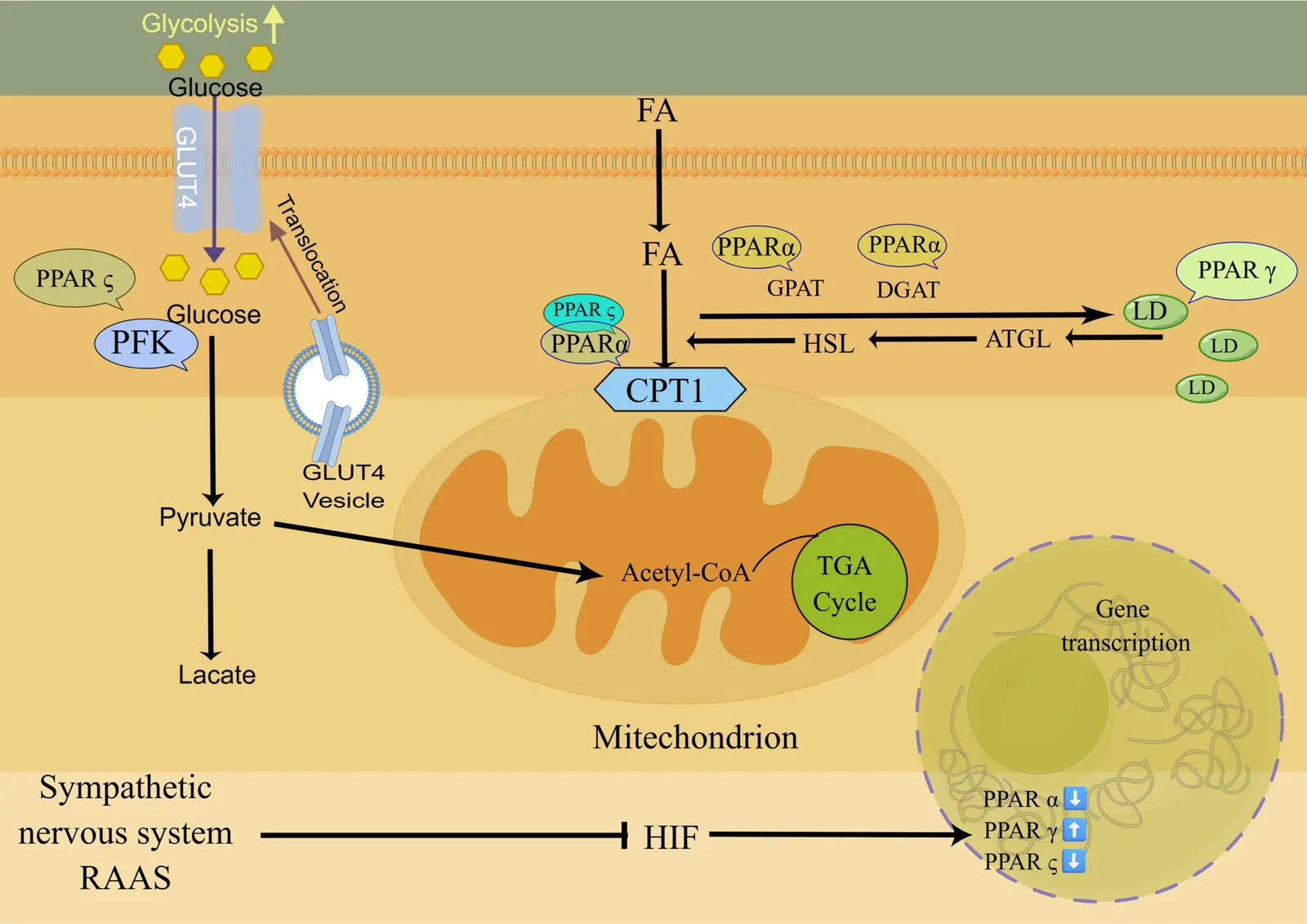
Tissue-specific regulation of energy metabolism by PPAR subtypes. The diagram illustrates the primary metabolic pathways controlled by PPARα (left), PPARβ/δ (center), and PPARγ (right) in their major tissues of action: liver, skeletal muscle, and adipose tissue, respectively. **Abbreviations:** PFK, phosphofructokinase; GPAT, glycerol-3-phosphate acyltransferase; HSL, hormone-sensitive lipase; DGAT, diacylglycerol O-acyltransferase; ATGL, adipose triglyceride lipase; CPT1, carnitine palmitoyl transferase I; TCA cycle, tricarboxylic acid cycle; GLUT4, glucose transporter type 4; LD, lipid droplet; FA, fatty acid; TG, triglyceride.

**Table 1:** Comparison of PPAR subtypes (PPARα, PPARβ/δ, PPARγ).

**PPAR** **Subtype**	**Gene** **Location**	**Ligand** **Types**	**Tissue** **Distribution**	**Related** **Diseases**	**Agonists/** **Antagonists**	**Pharmacological Features**	**Reference**
**PPARα**	Human chromosome 22q12	**Endogenous**: Fatty acids **Synthetic agonists**: Fenofibrate, Gemfibrozil	Liver, brown adipose tissue, kidney, heart	1. Metabolic syndrome (hyperlipidemia) 2. Cardiovascular diseases 3. Nonalcoholic fatty liver disease (NAFLD)	Agonists: Fibrates Antagonist: GW6471	Mainly used to treat hypertriglyceridemia	[10-13]
**PPARβ/δ (PPARδ)**	Human chromosome 6p21.3	**Endogenous**: Fatty acids B16 **Synthetic agonists**: GW501516, AGN194204	Widely expressed in almost all tissues	1. Metabolic diseases (*e.g*., obesity, diabetes) 2. Inflammatory diseases (*e.g*., psoriasis) 3. Cancers (*e.g*., skin cancer, colon cancer)	Agonists: Used in metabolic enhancement and antiinflammatory research Antagonist: GSK3787	Potential for treating metabolic diseases and aging-related disorders	[14-16]
**PPARγ**	Human chromosome 3p25	**Endogenous**: Prostaglandin D2 metabolites **Synthetic agonists**: Rosiglitazone, Pioglitazone	Adipose tissue (white/brown fat)	1. Type 2 diabetes 2. Obesity 3. Atherosclerosis	Agonists: Thiazolidinediones Antagonist: T0070907	Improves insulin resistance but may cause edema and weight gain	[6-9]

**Table 2:** Comparison of FABP subtypes (FABP1, 2, 3, 4, 8, 9, 10) : Tissue distribution, main functions and related diseases.

**Subtype**	**Tissue Distribution**	**Main Functions**	**Related Diseases**	**Reference**
**FABP1**	Liver, proximal tubules of kidney, small intestine	1.Involved in fatty acid transport and β-oxidation 2.Regulates gluconeogenesis and lipid metabolism 3.Antioxidative stress	Non-alcoholic fatty liver disease (NAFLD), liver cancer	[58, 60]
**FABP2**	Small intestinal epithelial cells (especially duodenum, jejunum)	1.Promotes intestinal fatty acid absorption and esterification 2.Regulates de novo lipid synthesis 3.Maintains intestinal barrier function	Obesity, metabolic syndrome, inflammatory bowel disease (IBD)	[56, 57]
**FABP3**	Cardiomyocytes, skeletal muscle, renal medulla	1.Cardiac energy metabolism (fatty acid transport to mitochondria) 2.Biomarker for myocardial injury (elevated levels in blood)	Myocardial infarction, heart failure, diabetic cardiomyopathy	[61]
**FABP4**	Adipose tissue (especially macrophages, adipocytes)	1. Lipid storage and inflammatory signaling in adipocytes 2.Regulates insulin resistance and metabolic syndrome 3.Involved in atherosclerosis	Obesity, type 2 diabetes, atherosclerosis, cancer	[59, 60, 62]
**FABP8**	Skeletal muscle, cardiomyocytes	1.Skeletal muscle fatty acid oxidation for energy supply 2.Energy metabolism regulation under exercise stress	Obesityrelated myopathy, metabolic syndrome	[60]
**FABP9**	Adipose tissue, macrophages, vascular endothelial cells	1.Regulates fatty acid transport to vascular endothelium 2.Involved in vascular inflammation and atherosclerosis	Atherosclerosis, metabolic syndrome	[60]
**FABP10**	Liver, intestinal tract	1.Involved in enterohepatic circulation of longchain fatty acids 2.Regulates apolipoprotein synthesis	Hyperlipidemia, obesity	[58]

## LIST OF ABBREVIATIONS

NAFLD: Non-Alcoholic Fatty Liver Disease
GLP-1: Glucagon-Like Peptide-1
SGLT2: Sodium-Glucose Cotransporter 2
FABP: Fatty Acid-Binding Protein
AMPK: Adenosine Monophosphate-Activated Protein Kinase
UCP: Uncoupling Protein
SCFAs: Short-Chain Fatty Acids
FXR: Farnesoid X Receptor
TGR5: G Protein-Coupled Bile Acid Receptor 1
IPA: Indolepropionic Acid
AHR: Aryl Hydrocarbon Receptor
DPP-4i: Dipeptidyl Peptidase-4 Inhibitors
MASLD: Metabolic Dysfunction-Associated Steatotic Liver Disease
MASH: Metabolic Dysfunction-Associated Steatohepatitis
CRISPR-Cas9: Clustered Regularly Interspaced Short Palindromic Repeats-CRISPR-Associated Protein 9

## References

[1] Zhang H., Zhou X.D., Shapiro M.D., Lip G.Y.H., Tilg H., Valenti L., Somers V.K., Byrne C.D., Targher G., Yang W., Viveiros O., Opio C.K., Mantzoros C.S., Ryan J.D., Kok K.Y.Y., Jumaev N.A., Perera N., Robertson A.G., Abu-Abeid A., Misra A., Wong Y.J., Ruiz-Úcar E., Ospanov O., Kızılkaya M.C., Luo F., Méndez-Sánchez N., Zuluaga M., Lonardo A., Al Momani H., Toro-Huamanchumo C.J., Adams L., Al-Busafi S.A., Sharara A.I., Chan W.K., Abbas S.I., Sookoian S., Treeprasertsuk S., Ocama P., Alswat K., Kong A.P.S., Ataya K., Lim-Loo M.C., Oviedo R.J., Szepietowski O., Fouad Y., Zhang H., Abdelbaki T.N., Katsouras C.S., Prasad A., Thaher O., Ali A., Molina G.A., Sung K.C., Chen Q.F., Lesmana C.R.A., Zheng M.H. (2024). Global burden of metabolic diseases, 1990–2021.. Metabolism.

[2] Maltese G., Koufakis T., Kotsa K., Basile G., Siow R. (2023). Mediterranean diet, type 2 diabetes prevention and healthy ageing: Do we need more evidence?. Diabetes Res. Clin. Pract..

[3] Zhang X., Liu J., Ni Y., Yi C., Fang Y., Ning Q., Shen B., Zhang K., Liu Y., Yang L., Li K., Yong L., Huang R., Li Z. (2024). Global prevalence of overweight and obesity in children and adolescents.. JAMA Pediatr..

[4] Le M.H., Yeo Y.H., Zou B., Barnet S., Henry L., Cheung R., Nguyen M.H. (2022). Forecasted 2040 global prevalence of nonalcoholic fatty liver disease using hierarchical bayesian approach.. Clin. Mol. Hepatol..

[5] Christofides A., Konstantinidou E., Jani C., Boussiotis V.A. (2021). The role of peroxisome proliferator-activated receptors (PPAR) in immune responses.. Metabolism.

[6] Nicchio I.G., Cirelli T., Quil L.C.C., Camilli A.C., Scarel-Caminaga R.M., Leite F.R.M. (2025). Understanding the peroxisome proliferator-activated receptor gamma (PPAR-γ) role in periodontitis and diabetes mellitus: A molecular perspective.. Biochem. Pharmacol..

[7] Shao X., Wang M., Wei X., Deng S., Fu N., Peng Q., Jiang Y., Ye L., Xie J., Lin Y. (2016). Peroxisome proliferator-activated receptor-γ: master regulator of adipogenesis and obesity.. Curr. Stem Cell Res. Ther..

[8] Maciejewska-Skrendo A., Massidda M., Tocco F., Leźnicka K. (2022). The influence of the differentiation of genes encoding peroxisome proliferator-activated receptors and their coactivators on nutrient and energy metabolism.. Nutrients.

[9] Ding H., Dong J., Wang Y., Huang Q., Xu J., Qiu Z., Yao F. (2023). Ginsenoside Rb1 interfered with macrophage activation by activating PPARγ to inhibit insulin resistance in obesity.. Molecules.

[10] Mal S., Dwivedi A.R., Kumar V., Kumar N., Kumar B., Kumar V. (2021). Role of peroxisome proliferator-activated receptor gamma (PPARγ) in different disease states: Recent updates.. Curr. Med. Chem..

[11] Han L., Shen W.J., Bittner S., Kraemer F.B., Azhar S. (2017). PPARs: Regulators of metabolism and as therapeutic targets in cardiovascular disease. Part I: PPAR-α.. Future Cardiol..

[12] Bhattacharjee J., Borra V.J., Salem E.S.B., Zhang C., Murakami K., Gill R.K., Kim A., Kim J.K., Salazar-Gonzalez R.M., Warren M., Kohli R., Nakamura T. (2021). Hepatic ago2 regulates pparα for oxidative metabolism linked to glycemic control in obesity and post bariatric surgery.. Endocrinology.

[13] Kersten S., Stienstra R. (2017). The role and regulation of the peroxisome proliferator activated receptor alpha in human liver.. Biochimie.

[14] Bougarne N., Weyers B., Desmet S.J., Deckers J., Ray D.W., Staels B., De Bosscher K. (2018). Molecular actions of PPARα in lipid metabolism and inflammation.. Endocr. Rev..

[15] Derosa G., Sahebkar A., Maffioli P. (2018). The role of various peroxisome proliferator‐activated receptors and their ligands in clinical practice.. J. Cell. Physiol..

[16] Hong F., Pan S., Guo Y., Xu P., Zhai Y. (2019). PPARs as Nuclear Receptors for Nutrient and Energy Metabolism.. Molecules.

[17] Wang Y., Nakajima T., Gonzalez F.J., Tanaka N. (2020). PPARs as Metabolic Regulators in the Liver: Lessons from Liver-Specific PPAR-Null Mice.. Int. J. Mol. Sci..

[18] Teimouri M., Hosseini H., ArabSadeghabadi Z., Babaei-Khorzoughi R., Gorgani-Firuzjaee S., Meshkani R. (2022). The role of protein tyrosine phosphatase 1B (PTP1B) in the pathogenesis of type 2 diabetes mellitus and its complications.. J. Physiol. Biochem..

[19] Hussain H., Green I.R., Abbas G., Adekenov S.M., Hussain W., Ali I. (2019). Protein tyrosine phosphatase 1B (PTP1B) inhibitors as potential anti-diabetes agents: Patent review (2015-2018).. Expert Opin. Ther. Pat..

[20] Prabhakar P.K., Sivakumar P.M. (2019). Protein tyrosine phosphatase 1b inhibitors: A novel therapeutic strategy for the management of type 2 diabetes mellitus.. Curr. Pharm. Des..

[21] Dodd G.T., Xirouchaki C.E., Eramo M., Mitchell C.A., Andrews Z.B., Henry B.A., Cowley M.A., Tiganis T. (2019). Intranasal targeting of hypothalamic PTP1B and TCPTP reinstates leptin and insulin sensitivity and promotes weight loss in obesity.. Cell Rep..

[22] Li M., Li H., Min X., Sun J., Liang B., Xu L., Li J., Wang S.H., Xu X. (2024). Identification of 1,3,4-thiadiazolyl-containing thiazolidine-2,4-dione derivatives as novel ptp1b inhibitors with antidiabetic activity.. J. Med. Chem..

[23] Myers R.W., Guan H.P., Ehrhart J., Petrov A., Prahalada S., Tozzo E., Yang X., Kurtz M.M., Trujillo M., Gonzalez Trotter D., Feng D., Xu S., Eiermann G., Holahan M.A., Rubins D., Conarello S., Niu X., Souza S.C., Miller C., Liu J., Lu K., Feng W., Li Y., Painter R.E., Milligan J.A., He H., Liu F., Ogawa A., Wisniewski D., Rohm R.J., Wang L., Bunzel M., Qian Y., Zhu W., Wang H., Bennet B., LaFranco Scheuch L., Fernandez G.E., Li C., Klimas M., Zhou G., van Heek M., Biftu T., Weber A., Kelley D.E., Thornberry N., Erion M.D., Kemp D.M., Sebhat I.K. (2017). Systemic pan-AMPK activator MK-8722 improves glucose homeostasis but induces cardiac hypertrophy.. Science.

[24] Yan S., Lu W., Zhou J., Guo X., Li J., Cheng H., Zhu X., Zhao Y., Duan M., Yang H., Zhang Y., Wang Q., Chen L., Zheng T. (2022). Aqueous extract of *Scrophularia ningpoensis* improves insulin sensitivity through AMPK-mediated inhibition of the NLRP3 inflammasome.. Phytomedicine.

[25] Canbolat E., Cakıroglu F.P. (2023). The importance of AMPK in obesity and chronic diseases and the relationship of AMPK with nutrition: A literature review.. Crit. Rev. Food Sci. Nutr..

[26] Belli I., Yaman M. (2020). the role of AMPK in the regulation of appetite and energy homeostasis: Role of AMPK in appetite.. International Journal of Innovative Research and Reviews.

[27] Lee J.M., Choi S.S., Park M.H., Jang H., Lee Y.H., Khim K.W., Oh S.R., Park J., Ryu H.W., Choi J.H. (2020). *Broussonetia papyrifera* root bark extract exhibits anti-inflammatory effects on adipose tissue and improves insulin sensitivity potentially *via* AMPK activation.. Nutrients.

[28] Song H., Cao Y., Huang Q. (2025). The polysaccharide PPS3 from pumpkin alleviates high fat diet-induced obesity in mice by activating the AMPK signaling pathway and regulating the gut microbiota.. Mol. Nutr. Food Res..

[29] Bao R., Meng Y., Zhang H., Yang C., Li W., Zhang C., Zhang J., Sun R., Li Z., Jiang W., Zhang C., Zhang C., Yuan H.X., Dang Y. (2021). Elaiophylin reduces body weight and lowers glucose levels in obese mice by activating AMPK.. Cell Death Dis..

[30] Min S.H., Song D.K., Lee C.H., Roh E., Kim M.S. (2024). Hypothalamic AMP-activated protein kinase as a whole-body energy sensor and regulator.. Endocrinol. Metab..

[31] Tarasiuk O., Miceli M., Di Domizio A., Nicolini G. (2022). AMPK and diseases: State of the art regulation by AMPK-targeting molecules.. Biology.

[32] Čater M., Križančić Bombek L.K. (2022). Protective role of mitochondrial uncoupling proteins against age-related oxidative stress in type 2 diabetes mellitus.. Antioxidants.

[33] Jalloul W., Grierosu I.C., Jalloul D., Stefanescu C., Ghizdovat V. (2025). Functional complexity of thermogenic adipose tissue: From thermogenesis to metabolic and fibroinflammatory crosstalk.. Int. J. Mol. Sci..

[34] Jones S.A., Ruprecht J.J., Crichton P.G., Kunji E.R.S. (2024). Structural mechanisms of mitochondrial uncoupling protein 1 regulation in thermogenesis.. Trends Biochem. Sci..

[35] Matta L., Blaas L., de Faria C.C. (2023). From honeymoon to dysfunction: Brown fat remodelling in obesity.. J. Physiol..

[36] Jeong H.W., Choi R.H., Koh H.J. (2021). Obesity-induced TRB3 negatively regulates Brown adipose tissue function in mice.. Biochem. Biophys. Res. Commun..

[37] Luby A., Alves-Guerra M.C. (2022). UCP2 as a cancer target through energy metabolism and oxidative stress control.. Int. J. Mol. Sci..

[38] Hao L., Li S., Chen G., Hu X. (2024). Regulation of UCP2 in nonalcoholic fatty liver disease: From mechanisms to natural product.. Chem. Biol. Drug Des..

[39] Din I., Majid S., Rashid F., Wani M.D., Qadir J., Wani H., Fareed M. (2023). Mitochondrial uncoupling protein 2 (UCP2) gene polymorphism − 866 G/A in the promoter region is associated with type 2 diabetes mellitus among Kashmiri population of Northern India.. Mol. Biol. Rep..

[40] Kim D.H., Kim H.J., Seong J.K. (2022). UCP2 KO mice exhibit ameliorated obesity and inflammation induced by high-fat diet feeding.. BMB Rep..

[41] Della Guardia L., Luzi L., Codella R. (2024). Muscle-UCP3 in the regulation of energy metabolism.. Mitochondrion.

[42] Park S.S., Seo Y.K. (2020). Excess accumulation of lipid impairs insulin sensitivity in skeletal muscle.. Int. J. Mol. Sci..

[43] Alcalá M., Calderon-Dominguez M., Serra D., Herrero L., Viana M. (2019). Mechanisms of impaired brown adipose tissue recruitment in obesity.. Front. Physiol..

[44] Zhu X., Zhang X., Gao X., Yi Y., Hou Y., Meng X., Jia C., Chao B., Fan W., Li X., Zhang H. (2020). Effects of inulin propionate ester on obesity-related metabolic syndrome and intestinal microbial homeostasis in diet-induced obese mice.. ACS Omega.

[45] Yang M., Cai W., Li X., Deng Y., Li J., Wang X., Zhu L., Wang C., Xiaoqiong L. (2024). The effect of type 2 resistant starch and indole-3-propionic acid on ameliorating high-fat-diet-induced hepatic steatosis and gut dysbiosis.. Foods.

[46] Jung T.W., Park J., Sun J.L., Ahn S.H., Abd El-Aty A.M., Hacimuftuoglu A., Kim H.C., Shim J.H., Shin S., Jeong J.H. (2020). Administration of kynurenic acid reduces hyperlipidemia-induced inflammation and insulin resistance in skeletal muscle and adipocytes.. Mol. Cell. Endocrinol..

[47] Traisaeng S., Batsukh A., Chuang T.H., Herr D.R., Huang Y.F., Chimeddorj B., Huang C.M. (2020). Leuconostoc mesenteroides fermentation produces butyric acid and mediates Ffar2 to regulate blood glucose and insulin in type 1 diabetic mice.. Sci. Rep..

[48] Li Y., Wang L., Yi Q., Luo L., Xiong Y. (2024). Regulation of bile acids and their receptor FXR in metabolic diseases.. Front. Nutr..

[49] Yu H., Nie R., Shen C. (2023). The role of bile acids in regulating glucose and lipid metabolism.. Endocr. J..

[50] Chiang J.Y.L., Ferrell J.M. (2022). Discovery of farnesoid X receptor and its role in bile acid metabolism.. Mol. Cell. Endocrinol..

[51] Ferrell J.M., Dilts M., Stahl Z., Pokhrel S., Boehme S., Chiang J.Y.L. (2022). The bile acid receptor tgr5 and high fat, high sugar-induced liver injury.. FASEB J..

[52] Luxenburger A., Harris L.D., Ure E.M., Jiao W., Woolhouse A.D., Cameron S.A., Weymouth-Wilson A., Furneaux R.H., Pitman J.L., Hinkley S.F.R. (2023). The discovery of 12β-methyl-17-epi-18-nor-bile acids as potent and selective TGR5 agonists.. Eur. J. Med. Chem..

[53] Wang Z., Yang S., Liu L., Mao A., Kan H., Yu F., Ma X., Feng L., Zhou T. (2024). The gut microbiota-derived metabolite indole-3-propionic acid enhances leptin sensitivity by targeting STAT3 against diet-induced obesity.. Clin. Transl. Med..

[54] Huang T., Song J., Gao J., Cheng J., Xie H., Zhang L., Wang Y.H., Gao Z., Wang Y., Wang X., He J., Liu S., Yu Q., Zhang S., Xiong F., Zhou Q., Wang C.Y. (2022). Adipocyte-derived kynurenine promotes obesity and insulin resistance by activating the AhR/STAT3/IL-6 signaling.. Nat. Commun..

[55] Liu J., Li F., Yang L., Luo S., Deng Y. (2025). Gut microbiota and its metabolites regulate insulin resistance: Traditional Chinese medicine insights for T2DM.. Front. Microbiol..

[56] Du W., Jiang S., Yin S., Wang R., Zhang C., Yin B.C., Li J., Li L., Qi N., Zhou Y., Ye B.C. (2024). The microbiota-dependent tryptophan metabolite alleviates high-fat diet–induced insulin resistance through the hepatic AhR/TSC2/mTORC1 axis.. Proc. Natl. Acad. Sci..

[57] Niu Y., Hu X., Song Y., Wang C., Luo P., Ni S., Jiao F., Qiu J., Jiang W., Yang S., Chen J., Huang R., Jiang H., Chen S., Zhai Q., Xiao J., Guo F. (2024). *Blautia coccoides* is a newly identified bacterium increased by leucine deprivation and has a novel function in improving metabolic disorders.. Adv. Sci..

[58] Castan-Laurell I., Dray C., Valet P. (2021). The therapeutic potentials of apelin in obesity-associated diseases.. Mol. Cell. Endocrinol..

[59] Palmer E.S., Irwin N., O’Harte F.P.M. (2022). Potential therapeutic role for apelin and related peptides in diabetes: An update.. Clin. Med. Insights Endocrinol. Diabetes.

[60] Schinzari F., Veneziani A., Mores N., Barini A., Di Daniele N., Cardillo C., Tesauro M. (2017). Beneficial effects of apelin on vascular function in patients with central obesity.. Hypertension.

[61] Wen R., Huang R., Xu K., Cheng Y., Yi X. (2023). Beneficial effects of Apelin-13 on metabolic diseases and exercise.. Front. Endocrinol..

[62] Bertrand C., Pradère J.P., Geoffre N., Deleruyelle S., Masri B., Personnaz J., Le Gonidec S., Batut A., Louche K., Moro C., Valet P., Castan-Laurell I. (2018). Chronic apelin treatment improves hepatic lipid metabolism in obese and insulin-resistant mice by an indirect mechanism.. Endocrine.

[63] Singh A., Sohal A., Batta A. (2024). GLP-1, GIP/GLP-1, and GCGR/GLP-1 receptor agonists: Novel therapeutic agents for metabolic dysfunction-associated steatohepatitis.. World J. Gastroenterol..

[64] Njei B., Al-Ajlouni Y., Lemos S.Y., Ugwendum D., Ameyaw P., Njei L.P., Boateng S. (2024). Efficacy and safety of GLP-1 receptor agonists in patients with metabolic dysfunction-associated steatotic liver disease: A systematic review and meta-analysis of randomized controlled trials.. Cureus.

[65] Wang M. W., Lu L. G. (2025). Current status of glucagon-like peptide-1 receptor agonists in metabolic dysfunction-associated steatotic liver disease: A clinical perspective.. J. Clin. Transl. Hepatol..

[66] Soresi M., Giannitrapani L. (2024). Glucagon-like peptide 1 agonists are potentially useful drugs for treating metabolic dysfunction-associated steatotic liver disease.. World J. Gastroenterol..

[67] Melzer-Cohen C., Chodick G., Husemoen L.L.N., Rhee N., Shalev V., Karasik A. (2019). A retrospective database study of liraglutide persistence associated with glycemic and body weight control in patients with type 2 diabetes.. Diabetes Ther..

[68] Butt M.D., Ong S.C., Rafiq A., Batool N., Saifi R., Yaseen S., Kaukab I., Ramzan B. (2024). Long-Term clinical efficacy of liraglutide for type 2 diabetes: Real-world evidence and outcomes from Pakistan.. J. Pharm. Policy Pract..

[69] Gentilella R., Sesti G., Vazquez L., Sapin H., Reed V., Romera I., Pozzilli P. (2019). Dulaglutide is an effective treatment for lowering HbA1c in patients with type 2 diabetes regardless of body mass index.. Diabetes Obes. Metab..

[70] Saisho Y. (2020). SGLT2 Inhibitors: The Star in the Treatment of Type 2 Diabetes?. Diseases.

[71] Tian Y., Zhou C., Yan Q., Li Z., Chen D., Feng B., Song J. (2025). Dapagliflozin improves diabetic kidney disease by inhibiting ferroptosis through β-hydroxybutyrate production.. Ren. Fail..

[72] Chauhan S., Bhandari U., Habib A. (2025). Efficacy of dapagliflozin and telmisartan combination therapy in reducing albuminuria and inflammatory markers in diabetic nephropathy: A prospective observational study.. Curr. Vasc. Pharmacol..

[73] Filippas-Ntekouan S., Dimou A., Dafopoulos P., Kostara C., Bairaktari E., Chasapi S., Spyroulias G., Koufakis T., Koutsovasilis A., Tsimihodimos V. (2024). Effect of dapagliflozin on the serum metabolome in patients with type 2 diabetes mellitus.. J. Diabetes Metab. Disord..

[74] Yokomizo H., Kawanami D., Sonoda N., Ono Y., Maeda Y., Itoh J., Tohyama T., Hirose M., Watanabe H., Kishimoto J., Ogawa Y., Inoguchi T. (2025). Relationship of dietary intake and eating behaviors with glycemic control and body weight under long-term treatment with dapagliflozin: An exploratory prospective study.. Diabetol. Int..

[75] Leońska-Duniec A., Świtała K., Ahmetov I.I., Pickering C., Massidda M., Buryta M., Mastalerz A., Maculewicz E. (2021). *FABP2* ala54thr polymorphism and post-training changes of body composition and biochemical parameters in caucasian women.. Genes.

[76] Yabut K.C.B., Isoherranen N. (2023). Impact of intracellular lipid binding proteins on endogenous and xenobiotic ligand metabolism and disposition.. Drug Metab. Dispos..

[77] Wang Z., Yue Y.X., Liu Z.M., Yang L.Y., Li H., Li Z.J., Li G.X., Wang Y.B., Tian Y.D., Kang X.T., Liu X.J. (2019). Genome-Wide analysis of the *FABP* gene family in liver of chicken (Gallus gallus): Identification, dynamic expression profile, and regulatory mechanism.. Int. J. Mol. Sci..

[78] Liu S., Wu D., Fan Z., Yang J., Li Y., Meng Y., Gao C., Zhan H. (2022). FABP4 in obesity-associated carcinogenesis: Novel insights into mechanisms and therapeutic implications.. Front. Mol. Biosci..

[79] Zhang Y., Zhang J., Ren Y., Lu R., Yang L., Nie G. (2020). Tracing the evolution of fatty acid-binding proteins (FABPs) in organisms with a heterogeneous fat distribution.. FEBS Open Bio.

[80] Nguyen H.C., Bu S., Nikfarjam S., Rasheed B., Michels D.C.R., Singh A., Singh S., Marszal C., McGuire J.J., Feng Q., Frisbee J.C., Qadura M., Singh K.K. (2023). Loss of fatty acid binding protein 3 ameliorates lipopolysaccharide-induced inflammation and endothelial dysfunction.. J. Biol. Chem..

[81] Trojnar M., Patro-Małysza J., Kimber-Trojnar Ż., Leszczyńska-Gorzelak B., Mosiewicz J. (2019). Associations between fatty acid-binding protein 4–a proinflammatory adipokine and insulin resistance, gestational and type 2 diabetes mellitus.. Cells.

[82] Yang S., Li S., Chang J. (2022). Discovery of Cobimetinib as a novel A-FABP inhibitor using machine learning and molecular docking-based virtual screening.. RSC Advances.

[83] Chen Y., Liao Z., Mao J., Wang W., Liu Y., Dai W., Wen Z., Liu S., Chen Y., Ma Y., Wang X., Li Z. (2025). Discovery of the first-in-class FABP/PPAR multiple modulator for the treatment of metabolic dysfunction-associated steatohepatitis.. Eur. J. Med. Chem..

[84] Beysen C., Schroeder P., Wu E., Brevard J., Ribadeneira M., Lu W., Dole K., O’Reilly T., Morrow L., Hompesch M., Hellerstein M.K., Li K., Johansson L., Kelly P.F. (2021). Inhibition of fatty acid synthase with FT-4101 safely reduces hepatic de novo lipogenesis and steatosis in obese subjects with non-alcoholic fatty liver disease: Results from two early‐phase randomized trials.. Diabetes Obes. Metab..

[85] Guo S., Yan K., Fang X., Ni Y., Ma W., Zhao R. (2021). α-lipoic acid alleviates hepatic lipid deposition by inhibiting FASN expression *via* miR-3548 in rats.. Nutrients.

[86] Zhang M., Tang Y., Tang E., Lu W. (2020). MicroRNA-103 represses hepatic de novo lipogenesis and alleviates NAFLD *via* targeting FASN and SCD1.. Biochem. Biophys. Res. Commun..

[87] Fan J., Li H., Nie X., Yin Z., Zhao Y., Chen C., Wang D.W. (2017). MiR-30c-5p ameliorates hepatic steatosis in leptin receptor-deficient (db/db) mice *via* down-regulating FASN.. Oncotarget.

[88] Li X., Zhang Z., Zhang M., Cao Y., Zhou W., Kou L., Guo W., Zhang B., Li S., Xu B. (2025). Mechanism of *O* ‐ GlcNAcylation regulating liver lipid synthesis in mice through FASN.. FASEB J..

[89] Cai C., Yu H., Huang G., Du X., Yu X., Zhou Y., Shen W. (2018). Histone modifications in fatty acid synthase modulated by carbohydrate responsive element binding protein are associated with non‑alcoholic fatty liver disease.. Int. J. Mol. Med..

[90] Xie Y., Shen F., He Y., Guo C., Yang R., Cao H., Pan Q., Fan J. (2023). Gamma-muricholic acid inhibits nonalcoholic steatohepatitis: Abolishment of steatosis-dependent peroxidative impairment by FXR/SHP/LXRα/FASN signaling.. Nutrients.

[91] Bołdys A., Bułdak Ł., Maligłówka M., Surma S., Okopień B. (2023). Potential therapeutic strategies in the treatment of metabolic-associated fatty liver disease.. Medicina.

[92] Kang S.M., Park J.H. (2021). Pleiotropic benefits of DPP-4 inhibitors beyond glycemic control.. Clin. Med. Insights Endocrinol. Diabetes.

[93] Almagthali A. G., Alkhaldi E. H., Alzahrani A. S., Alghamdi A. K., Alghamdi W. Y., Kabel A. M. (2019). Dipeptidyl peptidase-4 inhibitors: Anti-diabetic drugs with potential effects on cancer.. Diabetes Metab. Syndr..

[94] Ahrén B. (2021). Glucose-lowering action through targeting islet dysfunction in type 2 diabetes: Focus on dipeptidyl peptidase-4 inhibition.. J. Diabetes Investig..

[95] Adhikari J., Hirai T., Kawakita E., Iwai K., Koya D., Kanasaki K. (2023). Linagliptin ameliorated cardiac fibrosis and restored cardiomyocyte structure in diabetic mice associated with the suppression of necroptosis.. J. Diabetes Investig..

[96] Singh A.K., Yadav D., Sharma N., Jin J.O. (2021). Dipeptidyl peptidase (DPP)-IV inhibitors with antioxidant potential isolated from natural sources: A novel approach for the management of diabetes.. Pharmaceuticals.

[97] Alsalim W., Göransson O., Tura A., Pacini G., Mari A., Ahrén B. (2020). Persistent whole day meal effects of three dipeptidyl peptidase-4 inhibitors on glycaemia and hormonal responses in metformin-treated type 2 diabetes.. Diabetes Obes. Metab..

[98] Bastin M., Andreelli F. (2019). Dual GIP–GLP1-receptor agonists in the treatment of type 2 diabetes: A short review on emerging data and therapeutic potential.. Diabetes Metab. Syndr. Obes..

[99] Baggio L.L., Drucker D.J. (2021). Glucagon-like peptide-1 receptor co-agonists for treating metabolic disease.. Mol. Metab..

[100] Lahey A.M., Duprey K., Montague R.C., Schadler A.D., Naseman K.W. (2025). Insulin requirements after switching from GLP-1 receptor agonist to dual GIP/GLP-1 receptor agonist in patients with type 2 diabetes mellitus.. Pharmacotherapy.

[101] Min J.S., Jo S.J., Lee S., Kim D.Y., Kim D.H., Lee C.B., Bae S.K. (2025). A comprehensive review on the pharmacokinetics and drug−drug interactions of approved GLP-1 receptor agonists and a dual GLP-1/GIP receptor agonist.. Drug Des. Devel. Ther..

[102] Liu Q.K. (2024). Mechanisms of action and therapeutic applications of GLP-1 and dual GIP/GLP-1 receptor agonists.. Front. Endocrinol..

[103] Mu Y., Bao X., Eliaschewitz F.G., Hansen M.R., Kim B.T., Koroleva A., Ma R.C.W., Yang T., Zu N., Liu M., STEP 7 Study Group (2024). Efficacy and safety of once weekly semaglutide 2·4 mg for weight management in a predominantly east Asian population with overweight or obesity (STEP 7): A double-blind, multicentre, randomised controlled trial.. Lancet Diabetes Endocrinol..

[104] Gu W., Lu Y., Ye X., Yuan G., Liu D., Shen Z., Zu N., Mu Y. (2025). Efficacy and safety of once‐weekly semaglutide 2.4 mg for weight management in participants from China: A prespecified analysis of the STEP 7 randomized clinical trial.. Diabetes Obes. Metab..

[105] Frías J.P., Davies M.J., Rosenstock J., Pérez Manghi F.C., Fernández Landó L., Bergman B.K., Liu B., Cui X., Brown K., SURPASS-2 Investigators (2021). Tirzepatide *versus* Semaglutide Once Weekly in Patients with Type 2 Diabetes.. N. Engl. J. Med..

[106] Yamane S., Harada N., Inagaki N. (2025). Physiology and clinical applications of GIP.. Endocr. J..

[107] Zafer M., Tavaglione F., Romero-Gómez M., Loomba R. (2025). Review Article: GLP-1 Receptor Agonists and Glucagon/GIP/GLP-1 receptor dual or triple agonists—mechanism of action and emerging therapeutic landscape in MASLD. *Alimentary Pharmacology*. Therapeutics.

[108] Sadagopan A. (2023). Artificial intelligence driving diabetes care.. J. Int. Med. Grad. Stud..

[109] Wang B., Zhang T., Liu Q., Sutcharitchan C., Zhou Z., Zhang D., Li S. (2025). Elucidating the role of artificial intelligence in drug development from the perspective of drug-target interactions.. J. Pharm. Anal..

[110] Pan S., Yin L., Liu J., Tong J., Wang Z., Zhao J., Liu X., Chen Y., Miao J., Zhou Y., Zeng S., Xu T. (2024). Metabolomics-driven approaches for identifying therapeutic targets in drug discovery.. MedComm.

[111] Koodamvetty A., Thangavel S. (2025). Advancing precision medicine: Recent innovations in gene editing technologies.. Adv. Sci..

[112] Liu D., Cao D., Han R. (2025). Recent advances in therapeutic gene-editing technologies.. Mol. Ther..

[113] Borah A., Singh S., Chattopadhyay R., Kaur J., Bari V.K. (2024). Integration of CRISPR/Cas9 with multi-omics technologies to engineer secondary metabolite productions in medicinal plant: Challenges and Prospects.. Funct. Integr. Genomics.

[114] Liang C., Liu X., Sun Z., Wen L., Wu J., Shi S., Xiaolian L., Luo N., Li X. (2025). Lipid nanosystems for fatty liver therapy and targeted medication delivery: A comprehensive review.. Int. J. Pharm..

[115] Priya S., Desai V.M., Singhvi G. (2023). Surface modification of lipid-based nanocarriers: A potential approach to enhance targeted drug delivery.. ACS Omega.

